# Confocal microscopy reveals alterations of thylakoids in *Limnospira fusiformis* during prophage induction

**DOI:** 10.1007/s00709-021-01656-8

**Published:** 2021-05-02

**Authors:** Maryam Alsadat Zekri, Michael Schagerl, Johannes Schweichhart, Ingeborg Lang

**Affiliations:** Department of Functional and Evolutionary Ecology, University of Vienna, Althanstrasse 14, 1090 Vienna, Austria

**Keywords:** Flow cytometry, Confocal laser scanning microscopy, Cyanobacteria, Cyanophages, Mitomycin C, Photoautotroph, Microorganism

## Abstract

The alkaliphilic cyanobacterium *Limnospira fusiformis* is an integral part in food webs of tropical soda lakes. Recently, sudden breakdowns of *Limnospira* sp. blooms in their natural environment have been linked to cyanophage infections. We studied ultrastructural details and prophage components in the laboratory by means of confocal laser scanning microscopy (CLSM) and transmission electron microscopy (TEM). For a comparison at the subcellular level, we included transmission electron microscopy (TEM) material of infected cells collected during a field survey. Compared to TEM, CLSM has the advantage to rapidly providing results for whole, intact cells. Moreover, many cells can be studied at once. We chemically induced lysogenic cyanophages by means of mitomycin C (MMC) treatments and studied the ultrastructural alterations of host cells. In parallel, the number of cyanophages was obtained by flow cytometry. After treatment of the culture with MMC, flow cytometry showed a strong increase in viral counts, i.e., prophage induction. CLSM reflected the re-organization of *L. fusiformis* with remarkable alterations of thylakoid arrangements after prophage induction. Our study provides a first step towards 3D visualization of ultrastructure of cyanobacteria and showed the high potential of CLSM to investigate viral-mediated modifications in these groups.

## Introduction

The filamentous cyanobacterium *Limnospira fusiformis* (Voronichin) Nowicka-Krawczyk, Mühlsteinová & Hauer is one of the dominant photoautotrophs in tropical, saline alkaline lakes (Schagerl et al. 2015). Occasionally it is also found in temperate waters (Fužinato et al. 2010; Nowicka-Krawczyk et al. 2019). The filaments are unbranched and coiled. The helical shape varies within the species and is controlled by environmental factors such as temperature and irradiance supply (Gershwin and Belay 2007; Kaggwa et al. 2013a, b).

*Limnospira fusiformis* is a key element of food webs and any fluctuations in its density may affect other species communities (Kaggwa et al. 2013a, b; Krienitz and Kotut 2010; Kumssa and Bekele 2014). *Limnospira* spp. (trade name *Spirulina platensis*) is known for its high nutritional value and considered one of the most industrially cultivated photoautotrophic microorganisms (Vonshak and Tomaselli 2007). It is grown in large-scale cultivation systems and then used as a food supplement due to its high protein content (Seghiri et al. 2019) and other valuable compounds, which provide several health benefits (Arora Soni and Rana 2017).

Until recently, *L. fusiformis* was listed under the name *Arthrospira fusiformis*. The new genus *Limnospira* sp. was described based on phylogenetic analysis, ultrastructure, and ecological characteristics of *L. fusiformis* and *Arthrospira jenneri* Stizenberger ex Gomont (Nowicka-Krawczyk et al. 2019). The presence of a calyptra (a thickened cell wall in apical cells) and the arrangement of thylakoids of *Limnospira* are the main ultrastructural features that separate this taxon from *Arthrospira*. Moreover, thylakoids are arranged irregularly and form whirl-like (twisted) structures throughout the cells in *Limnospira*, while they are placed radially in *Arthrospira* (Nowicka-Krawczyk et al. 2019). Although the arrangement of thylakoids is more or less stable in certain phylogenetic clades and has been used for classification of cyanobacteria groups (Mareš et al. 2019), it may change also due to external factors.

Within the normal cell cycle, cyanobacterial DNA is expanding throughout the cell just before division. High-voltage cryo-electron tomography of *Synechococcus elongatus* showed condensation of DNA during light supply in the center of the cells (Murata et al. 2016). This synchronization of DNA condensation and cell division in *S. elongatus* with circadian rhythms indicates the dependence of DNA replication on photosynthetic electron transport (Ohbayashi et al. 2013). Nevertheless, compaction of DNA is difficult to distinguish as it is very transient, and the structure of DNA strongly changes during cell division. Sample preparation for electron microscopy, however, may cause artifacts and therefore live imaging seems to be a rational technique to avoid such pitfalls (Murata et al. 2016).

A few years back, cyanophage infections of *Limnospira* sp. were observed in both natural systems (Peduzzi et al. 2014) and large-scale cultivation units (Jacquet et al. 2013). So far as is known, cyanophages threatening cyanobacterial colonies belong to the tailed phages (Mann 2003). These phages consist of an icosahedral capsid containing the viral genome and a tail of variable length. In Myoviridae, the tail is long and contractile whereas Siphoviridae and Podoviridae have a non-contractile tail (Ackermann 1998). Tailed phages can cause either lytic or lysogenic infections in cyanobacteria⁠. While lytic cyanophages of marine *Synechococcus* sp. and *Prochlorococcus* sp. have been studied in detail (Puxty et al. 2016), only a few accounts of lysogenic cyanophages have been described so far (Cannon et al. 1971; Chu et al. 2011; Lee et al. 2006; Ohki and Fujita 1996). While lytic infections set off a continuous sequence resulting in viral reproduction and subsequent lysis of the host, lysogenic infections lead to the integration of the phage genome into the host genome as so-called prophage. Prophages can persist through several division cycles of the host until specific events trigger a transition to the lytic reproduction of the phage. Such an event is referred to as “induction” and can be mediated by DNA damage, e.g., through UV radiation, heavy metals like copper and cadmium (Lee et al. 2006; Singh et al. 2013), and mutagens like mitomycin C (MMC). MMC is a well-known DNA cross-linker, inhibiting host DNA synthesis (Barnett and Brundage 2010; Gad 2014; McKenna et al. 2012). MMC has been commonly used to induce prophages (Jacquet et al. 2013; Knowles et al. 2017; McDaniel et al. 2006; Paul 2008). The resulting lytic reproduction of phages turns the host into a “virocell” which displays altered physiological characteristics compared to an uninfected cell (Rosenwasser et al. 2016; Zimmerman et al. 2019)⁠. These physiological changes are triggered by the activation of the viral genome which brings about the manipulation of the host metabolism, the degradation of host DNA, and the intracellular formation of new viral particles or virions (Dai et al. 2013; Doron et al. 2016)⁠. Visible signs of these intracellular processes are usually investigated by transmission electron microscopy (TEM), but this technique is limited to the observation of virions inside of infected cells (Peduzzi et al. 2014). Recent studies relying on cryo-electron tomography (CET) allowed the observation of viral intermediates of lytic cyanophage infections in unprecedented detail (Dai et al. 2013) and demonstrated compartmentalization during infections of *Pseudomonas chlororaphis* phages (Chaikeeratisak et al. 2017). Previous work dealing with cyanobacteria and cyanophages showed additional ultrastructural changes, mainly involving rearrangements of thylakoid membranes such as invaginations (Sherman and Haselkorn 1970). Since these early studies, ultrastructural changes in cyanobacteria caused by cyanophages have hardly been studied.

We used confocal laser scanning microscopy (CLSM) to investigate the postulated changes within the host cells after phage induction. CLSM enables the study of living samples and even temporal changes in the ultrastructure of the organisms can be observed, for example in plasmolysis (Harant and Lang 2020) or cell division (Chan et al. 2005). By means of software packages, multi-dimensional views of image stacks can be assembled. The software facilitates spatial manipulation such as 3D reconstructions or rotations of the 3D image in arbitrary views and imaging degrees to provide a more comprehensive insight of structural components. We first induced the lytic cycle of cyanophages in *L. fusiformis* by addition of MMC to clone cultures, and then studied temporal and spatial changes in the distribution of DNA as well as alterations of thylakoids by means of CLSM. This way, we provide a method for fast screening of virus infections within the cyanobacterial host cells. We added TEM micrographs from a field study to complement the findings of CLSM. With this supplement, we intended to show the ultrastructure of *Limnospira* on a submicroscopical level and to show the mode of prophage development on the ultrastructural level.

## Material and methods

### Cultivation

*Limnospira fusiformis* originates from an algal culture farm in France. The unialgal culture was grown in Zarrouk medium (Morais et al. 2015) at 32 °C under a 20:4 h light–dark cycle (40 µmol photons m^−2^ s^−1^) and 100 rpm shaking. For MMC treatments, the culture was divided into two parts. In one flask, cyanobacteria were treated with MMC (final concentration 1.5 µg ml^−1^ culture); the other was used as a control. Thereafter, samples were taken at five time points (20, 22, 25, 28, 31 h) from both the MMC-treated culture and the control.

### Flow cytometry (FCM)

Three milliliters of sample was filtered through a syringe filter with 0.45 µm pore size. Then, 588 µl of filtered sample was fixed with 12 µl of 25% glutaraldehyde for 10 min. The fixed samples were immediately placed in liquid nitrogen for 30 min and stored at − 80 °C until analysis. Before FCM, the samples were diluted with 1 × Tris–EDTA buffer to achieve an event rate of 200–1200 events per second. After dilution, SYBR Green 1 of final concentration 0.5 × (Thermo Fisher Scientific) was added to each sample and then heated to 80 °C for exactly 10 min. Then the samples were immediately placed in ice water to cool down. The blank contained 1000 µl Tris–EDTA and 10 µl 50 × SYBR Green 1. Virus number was counted using a FACSAria III (Becton–Dickinson) FCM.

### Light microscopy and CLSM

Bright field images of the cyanobacterial habitus were taken from living cultures by means of light microscopes (Zeiss Axio-Imager, 20 × objective and Nikon Optiphot, 63 × oil immersion objective). For CLSM, 980 µl of the culture were fixed with 0.2% v/v glutaraldehyde and kept at room temperature in the dark until analysis. Microscopy was performed on a CLSM (Leica TCS SP5x/Leica DM 6000Cs) and using a 63 × water immersion objective. We used chlorophyll autofluorescence to study the thylakoid structure. The argon laser line of 496 nm was set at the intensity of 15% in combination with a 650–700 nm emission filter for chlorophyll examination of thylakoids. We applied 0.1 µg ml^−1^ DAPI (Molekula Group) diluted in phosphate-buffered saline (PBS) to stain DNA for CLSM. For DNA scrutiny with DAPI (Thermo Fisher Scientific), the UV diode (405 nm, 15% laser power) and a 420–480 nm long-pass filter were used. Five to ten minutes before microscopy, one drop of DAPI (0.1 µg/ml) was added to the sample directly on the slide. The scan speed was 400 ms^−1^ per pixel. The resolution was set to 1024 × 1024 pixel and the z steps were between 0.25 and 0.46 µm, adjusted with the number of individual images. Stack size was set to 20–30 images to reduce the bleaching during imaging and the time settings for all series were below 90 s. The gain was adjusted before each application to obtain optimum chlorophyll and DNA fluorescence. For 3D reconstruction and volume rendering, we used Amira software (6.2.0) with three series including 20 stacks each.

### Transmission electron microscopy (TEM)

Material was collected during a breakdown of a *Limnospira fusiformis* bloom in Lake Nakuru (Kenya). Filaments were concentrated in the field with a 30 µm plankton net and then immediately preserved with glutaraldehyde (final conc. 2.5%, EM-quality). Samples were then transported in ice-boxes to the laboratory (around 12 h). Glutaraldehyde was then replaced by 0.1 M Na-cacodylate buffer (three rinsing steps) and stored in the dark at 4 °C, because it took up to several weeks to transfer the samples to Austria for further preparation and analyses (otherwise the material would have become hard and brittle). Just before TEM studies, samples were fixed in glutaraldehyde for 15 h (final conc. 2.5%) and rinsed five times with MilliQ water, each rinsing step was 10 min and then transferred into osmium tetroxide for 2 h. After rinsing three times with MilliQ, each step 20 min, dehydration was performed with dimethoxypropane (DMP) for 15 min (Pernstich et al. 2003). Samples were then transferred into 100% acetone, with two rinsing steps of each 10 min, and then put into 100% acetonitrile for more 10 min (Edwards et al. 1992). The samples were then transferred into a 1:1 solution of acetonitrile and low viscosity resin (Agar Scientific, Stansted, and Essex, UK) for 3 h. Cyanobacteria were then transferred in pure resin for 4 h and then infiltrated at 40 °C for 12 h in in a cabinet dryer, followed by polymerization at 65 °C for 24 h. Ultra-thin sections of 70 nm were cut with a diamond knife (DiATOME, Biel-Bienne, Switzerland; Ultramicrotome Ultracut E, Reichert Jung, Austria) and placed on copper grids. They were treated with uranyl acetate for 30 min (0.5%), followed by lead citrate for 7 min (3%), at room temperature. Studies were done with a TEM Zeiss 902.

## Results

**Fig. 1:**
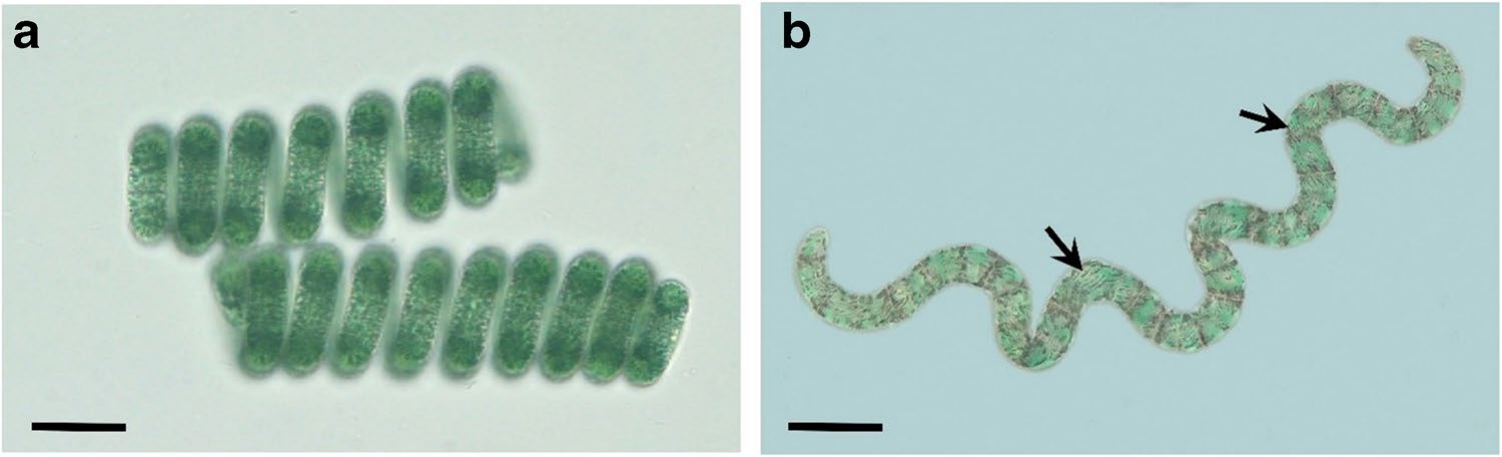
**a** Densely coiled filaments of *Limnospira fusiformis*. **b** A loosely coiled filament showing the characteristic gas vesicles (arrows). They appear almost black because of scattering and reflection. Scale bar = 25 µm

The filaments were characterized by the helicoid appearance (Fig. 1a). Gas vesicles, which facilitate buoyancy, appeared as grayish-blackish stripes (Fig. 1b). The color was caused by reflection and scattering effects.

**Fig. 2:**
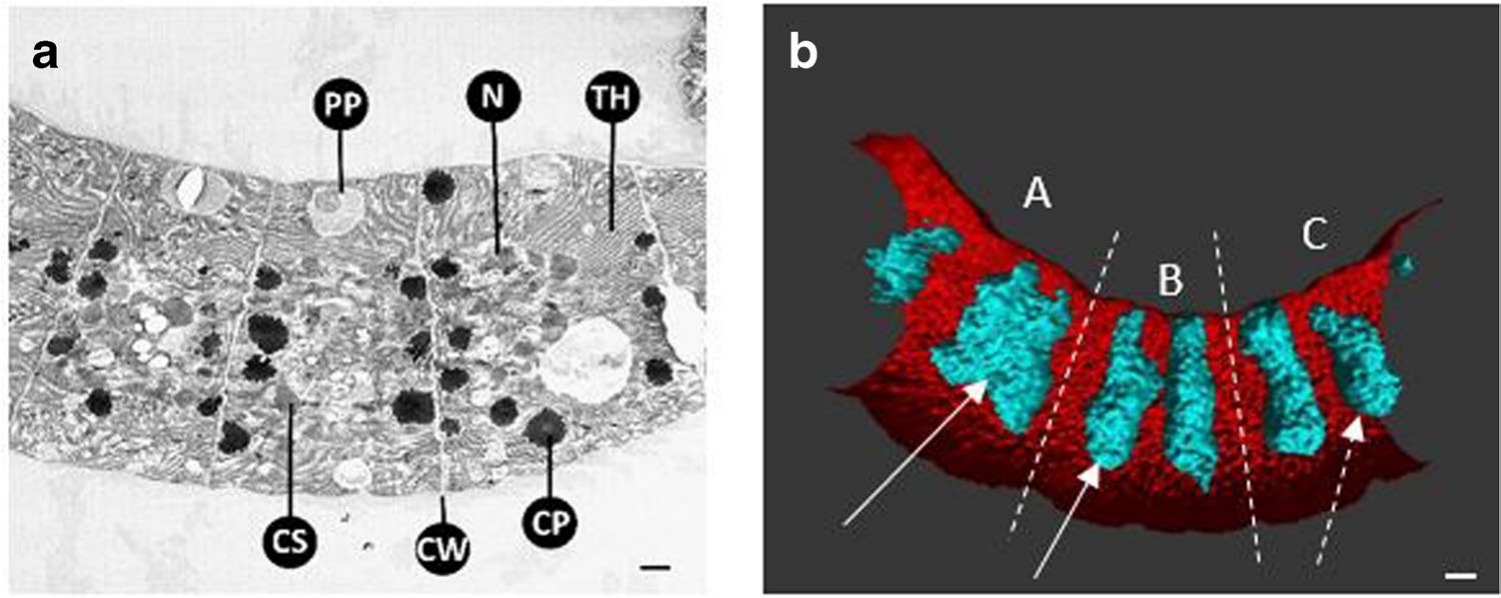
**a** TEM micrograph of a filament (longitudinal section). PP, polyphosphate (volutin) grains; N, condensed DNA; TH, thylakoids; CS, carboxysome; CW, cross wall; CP, cyanophycin granules (nitrogen storage) mainly located at the cross walls. **b** 3D visualization of thylakoids (red) and DNA (turquoise) in a filament showing different stages of cell division. Dashed lines indicate separate cells. Cell A and B: condensed DNA (solid arrow) with progressing separation. Cell C: cell division is almost completed and DNA separated (dashed arrow). Scale bar = 1 µm

Cells were characterized by irregular arrangement of thylakoids, forming a dense and irregular network of thylakoids (Fig. 2a). DNA appeared uniform and was located in the center of the cytoplasm, thylakoids were often located at the cell periphery (Fig. 2b), but sometimes also facing towards the center (Fig. 2a). Figure 2b showed a filament with cells at different division stages. DNA became compact just before cell division (cell A), and the DNA was compressed in the center of the cell. In cell B, DNA replication has been completed. In cell C, the formation of a new cell wall was already in progress.

**Fig. 3:**
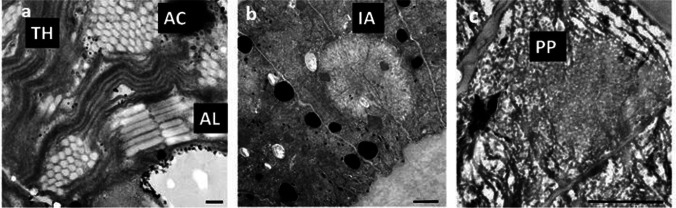
Ultrastructure of *Limnospira fusiformis* collected from Lake Nakuru (Kenya), photos M. & M. Gruber. **a** Detail of a healthy cell with thylakoids TH, gas vesicles (aerotopes) in cross-section AC and longitudinal section AL, scale bar 100 nm. **b** First signs of infection in a dividing cell, which appear as pale areas (“viroplasm”) IA, scale bar 1 µm. **c** Area with already developed capsids (gray dots) of prophages PP, scale bar 1 µm

TEM analysis from field material collected during a bloom crash showed distinct differences between healthy filaments (Fig. 3a) and infected cells (Fig. 3b, c). In healthy cells, thylakoids were irregularly arranged across cell lumen; gas vesicles could be observed in many cases and showed a characteristic pattern (Fig. 3a). The first visual signs of infection were spots of disintegrated cytoplasm, visible as pale areas with less discernable structures (Fig. 3b). During later stages, the formation of capsids of cyanophages became evident (Fig. 3c).

**Fig. 4:**
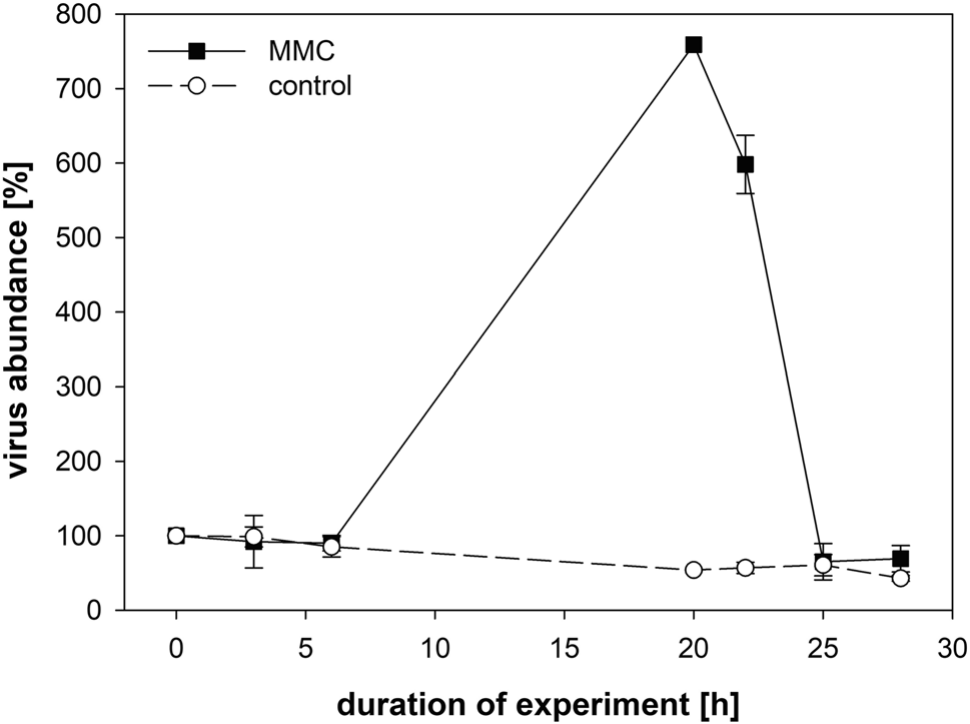
Number of viruses detected by flow cytometry treated with and without MMC at 7 time points. Solid line represents samples with MMC treatment; dashed line is the control (mean and standard error, *n* = 2)

FCM showed distinct prophage induction after treatment with MMC, as monitored by the viral outburst (Fig. 4). A steady increased in viral abundance occurred from 6 to 20 h in the MMC-treated cultures; the control did not show an increase in relative viral abundance (Fig. 4). The highest number of viruses was observed around 20 h after MMC treatment. The cultures started to change in color after 24 h. After 48 h, the cultures became pale indicating complete lysis of *L. fusiformis*.

**Fig. 5:**
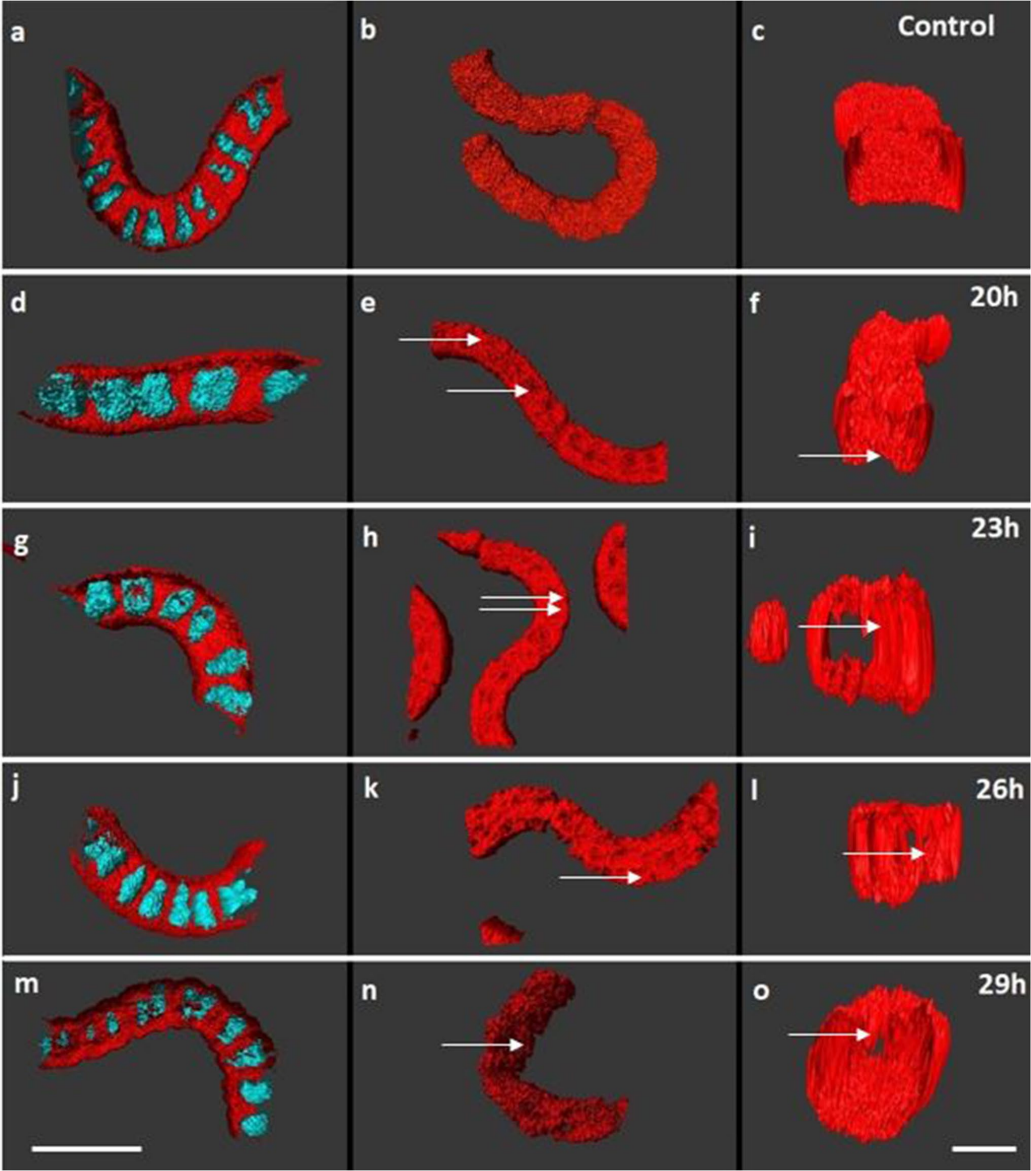
Confocal images and 3D reconstruction of *L. fusiformis* in control (**a**–**c**) and MMC-treated samples (**d**–**o**). **a**, **d**, **g**, **j**, **m** Overlay of stained DNA and thylakoids. **b**, **e**, **h**, **k**, **n** Thylakoids show invaginations as separations from the cell wall (arrows). **c**, **f**, **l**, **o** Reconstructed cross-sections of thylakoids within a filament cell reveal the progressive spread into the cell center. Arrows point the invagination in the thylakoids. Scale bar left column: 20 µm, scale bar right column: 5 µm

CLSM revealed the details of thylakoid alterations at time points 0, 20, 23, 26, and 29 h after phage induction. Figure 5 shows 3D reconstructions of whole filaments (left and center column) and their cross-sections (right column). The left column provides an overview of both, DNA and thylakoid fluorescence, the thylakoid structure alone is given in the middle column, and the right column shows the arrangements of thylakoids in a cross-section of the cells. At all time points, the DNA was arranged uniformly in the center of the cells with normal variations in compaction levels within cell division cycles (Fig. 5a, d, g, j, m; compare also Fig. 2b).

In the control, thylakoids were arranged irregularly next to the cell membrane and form a dense and intact network (Fig. 5b). In the cross-section, thylakoids were visible within the cell lumen and formed whirl-like structures in some areas (Fig. 5c). This pattern did not change throughout the whole experiment. Contrarily, distinct alterations were found in the MMC-treated culture. Visible alterations were observed from 20 h onwards, parallel to significantly increased virus number, and distinct invaginations in the thylakoids became visible (Fig. 5e, arrows). The cross-section at this time point showed a deformation of thylakoids in the form of invaginations and separations from the cell wall (Fig. 5f, arrow). This distinct pattern of strong thylakoid invaginations was still observed from 23 to 26 h after induction (Fig. 5h and k, arrows). Cross-sections revealed that in certain areas, thylakoids collapsed and formed a column-like structure pointing towards the center of the cell (Fig. 5i and l, arrows). After 29 h, thylakoids look significantly disturbed. Thylakoids were no longer continuously placed at the cell periphery and there was a disconnectivity throughout the network of thylakoids (Fig. 5n). The cross-section showed that thylakoids almost fill the center of the cell (Fig. 5o).

## Discussion

The ecological and commercial importance of *Limnospira* strongly call for detailed studies of virus-host interactions. To date, direct evidence for viral infections is obtained by means of TEM and CET, both being very time-consuming techniques that require several delicate preparation steps. Moreover, for whole cell studies, a series of ultrathin sections needs to be analyzed for cell reconstructions. The mean diameter of a filament is about 7 µm. For cutting a single cell, around 100 ultrathin sections are needed, and only a few of them will show capsid formation over a limited period of time. The probability of cutting an area of visible viroplasm is extremely low, because 1000 s of ultrathin sections must be screened to find a single visibly infected area. Exactly this drawback might be minimized in the future with the CLSM as an alternative and promising method for complementing ultrastructure studies. You first check for evidence/overall cyanophage patterns to enhance the chance for virus detection over time, and then you study details with TEM observations. Additionally, fluorescent markers allow for detailed, sub-structural analyses in a much larger sample size than in TEM.

There are only two published studies focusing on the submicroscopical level of cyanophage infections (Chu et al. 2011; Jin et al. 2019). For the genus-complex *Limnospira*/*Arthrospira*, two studies verified. Peduzzi et al. (2014) illustrated that *L. fusiformis* blooms are interrupted at irregular intervals through cyanophage infections. By means of TEM, they observed visible signs of viral infection in *L. fusiformis* cells. Jacquet et al. (2013) provided TEM photos of cyanopodivirus, which infected a culture originating from Peru. The correct nomenclature of the studied taxon is probably *L. maxima* (Voronichin) Nowicka-Krawczyk, Mühlsteinová & Hauer, because this taxon is commonly found in South America, but not in East Africa. To our knowledge, our study is the first report of successful induction of prophages of *L. fusiformis*.

Treatment with MMC was already successfully applied for prophage induction in various cyanobacteria like *Synechococcus* sp. (Chen et al. 2006). In our experiment, FCM measurements clearly demonstrated an increased abundance of viruses after MMC treatment. Although direct evidence is still lacking, the most likely cause for the observed increase in viral abundance is prophage induction from the culture, which also caused distinct changes in the ultrastructure of the thylakoid network. Samples for the visualization of the ultrastructure of *L. fusiformis* by CLSM were taken in accordance with the significant increase in virus abundance (Fig. 4). These samples most likely reflect the late stages of the replication cycle of the induced prophage which are coupled with the increased synthesis and subsequent release of phage progeny into the culture medium.

Based on CLSM images, the arrangement of thylakoids started to change 20 h after prophage induction in the form of invaginations of thylakoids towards the cell center. We conclude that these invaginations occur as a result of viral replication and assembly. “Viroplasm” is a large cytoplasmic area where viral replication and accumulation occur (Fig. 3b, c). Virus accumulation in the place of replication leads to the rearrangement of thylakoids and induces folding in the thylakoids. Our observation is in agreement with the study of Sherman and Haselkorn (1970), who reported an invagination of thylakoids in *Plectonema boryanum* after viral infection using TEM. We did not observe any alterations in thylakoids before 20 h, which is probably due to low initial frequency of prophages in the culture. The FCM experiment shows a peak of viral particles 20 h after prophage induction indicating cyanophage multiplication and release. The following sharp decrease in viral counts reflects the infection of the remaining *L. fusiformis* population. This amplification step is accompanied by a higher probability to observe the infected cells with ultrastructural modification. We did not focus on cell lysis, which is well studied in other cyanobacteria after cyanophage infection (Choi et al. 2010; Sherman and Haselkorn 1970).

CLSM has been applied successfully to visualize ultrastructural alterations of cyanobacteria in response to environmental factors (Murata et al. 2016) and to investigate DNA replication cycles (Manders et al. 1992). In the present study, DAPI labeling illustrated that different stages of cell division occur within the individual cells of a single *Limnospira* filament. We found no significant alterations in either DNA structure or DNA arrangement, which could be linked to cyanophage induction. We observed that the UV diode for DAPI excitation bleached the thylakoids during the time of scanning, caused greater signal intensity received from DNA. The strong signal resulted in observing unwanted objects and/or missing the details of DNA.

Recently, fascinating CET studies demonstrated that the reproduction of phages can lead to the formation of micro-compartments inside the host cells using microtubule-like filaments (Chaikeeratisak et al. 2019, 2017). These compartments have important functional roles including the protection against defense mechanisms of the host (Mendoza et al. 2020). To date such detailed observations are limited to the investigation of lytic phages. We induced the lysogenic cycle of the cyanophages by MMC and were able to visualize prophage induction by changes in thylakoid ultrastructure of the cyanobacterial host by means of CLSM. This method has the big advantage of immediate observation of live material. Changes in the range of minutes can be studied by optical sectioning and 3D reconstruction, which makes this method a promising tool for future studies of viral infections.
